# Forensic Psychiatric Outpatients’ and Therapists’ Perspectives on a Wearable Biocueing App (Sense-IT) as an Addition to Aggression Regulation Therapy: Qualitative Focus Group and Interview Study

**DOI:** 10.2196/40237

**Published:** 2023-02-01

**Authors:** Janna F ter Harmsel, Lisanne M Smulders, Matthijs L Noordzij, Lise T A Swinkels, Anna E Goudriaan, Arne Popma, Thimo M van der Pol

**Affiliations:** 1 Inforsa Forensic Mental Healthcare Amsterdam Netherlands; 2 Department of Child and Adolescent Psychiatry and Psychology Amsterdam Public Health Research Institute Amsterdam University Medical Center, Vrije Universiteit Amsterdam Amsterdam Netherlands; 3 Department of Psychology, Health and Technology University of Twente Enschede Netherlands; 4 Department of Research and Quality of Care Arkin Institute for Mental Health Amsterdam Netherlands; 5 Department of Psychiatry Amsterdam Public Health Research Institute Amsterdam University Medical Center, University of Amsterdam Amsterdam Netherlands; 6 Amsterdam Institute for Addiction Research Amsterdam Netherlands

**Keywords:** biocueing, biofeedback, aggression, behavior change, forensic psychiatry, wearable technology, mobile health, mHealth, implementation, mobile phone

## Abstract

**Background:**

Given the increased use of smart devices and the advantages of individual behavioral monitoring and assessment over time, wearable sensor–based mobile health apps are expected to become an important part of future (forensic) mental health care. For successful implementation in clinical practice, consideration of barriers and facilitators is of utmost importance.

**Objective:**

The aim of this study was to provide insight into the perspectives of both psychiatric outpatients and therapists in a forensic setting on the use and implementation of the Sense-IT biocueing app in aggression regulation therapy.

**Methods:**

A combination of qualitative methods was used. First, we assessed the perspectives of forensic outpatients on the use of the Sense-IT biocueing app using semistructured interviews. Next, 2 focus groups with forensic therapists were conducted to gain a more in-depth understanding of their perspectives on facilitators of and barriers to implementation.

**Results:**

Forensic outpatients (n=21) and therapists (n=15) showed a primarily positive attitude toward the addition of the biocueing intervention to therapy, with increased interoceptive and emotional awareness as the most frequently mentioned advantage in both groups. In the semistructured interviews, patients mainly reported barriers related to technical or innovation problems (ie, connection and notification issues, perceived inaccuracy of the feedback, and limitations in the ability to personalize settings). In the focus groups with therapists, 92 facilitator and barrier codes were identified and categorized into technical or innovation level (n=13, 14%), individual therapist level (n=28, 30%), individual patient level (n=33, 36%), and environmental and organizational level (n=18, 20%). The predominant barriers were limitations in usability of the app, patients’ motivation, and both therapists’ and patients’ knowledge and skills. Integration into treatment, expertise within the therapists’ team, and provision of time and materials were identified as facilitators.

**Conclusions:**

The chances of successful implementation and continued use of sensor-based mobile health interventions such as the Sense-IT biocueing app can be increased by considering the barriers and facilitators from patients’ and therapists’ perspectives. Technical or innovation-related barriers such as usability issues should be addressed first. At the therapist level, increasing integration into daily routines and enhancing affinity with the intervention are highly recommended for successful implementation. Future research is expected to be focused on further development and personalization of biocueing interventions considering what works for whom at what time in line with the trend toward personalizing treatment interventions in mental health care.

## Introduction

### Background

Over the last years, services that use information and communication strategies to improve and support health—eHealth—have grown tremendously owing to rapid technological changes [1,2]. In mental health care, a wide array of eHealth devices and programs is used, such as electronic patient records, internet-based therapy programs, and interventions using new technology (eg, virtual reality and serious gaming) [3-5]. More recently, mobile health (mHealth)—deploying smartphones and wearable devices to support health and health-related behaviors—has been added as a specific subcategory of eHealth [6,7].

Many eHealth interventions have been demonstrated to be feasible and acceptable [4]. Positive influences on health care outcomes and cost-effectiveness have been reported for specific populations [8]. However, to date, no firm conclusions can be drawn regarding the overall effectiveness of the use of eHealth in mental health care [1,4]. Concerning the use of mHealth interventions, overview studies identified several benefits, such as patient empowerment, self-monitoring, reduction of stigma, improved communication, and enhanced psychological services [7,9]. However, a recent review evaluating the usability of sensor-based mHealth apps reported insufficient acceptance by patients and recommended more rigorous research designs to investigate the effects of these particular interventions [10]. Therefore, the usability and clinical effectiveness of mHealth interventions need further assessment. Given the increasing use of smart devices and the advantages of individual behavioral monitoring and personal assessment over time, researchers expect mobile phone– and wearable sensor–based mHealth apps to become an increasingly important part of future personalized treatment [7,10].

In forensic psychiatry, personalized treatment interventions are highly relevant as a considerable number of forensic patients do not benefit from current treatment programs [11,12]. Limitations in effectivity might be related not only to characteristics associated with forensic populations, such as motivational difficulties and psychiatric complexity, but also to limited interoceptive awareness and insufficient transfer to out-of-session practice [13-15]. New technological interventions such as wearable biocueing apps might help overcome these challenges. Biocueing apps provide patients with real-time physiological feedback and just-in-time behavioral support messages when physiological tension increases, encouraging the use of adequate emotion regulation strategies in everyday life [16]. Another recent review supported the potential of eHealth for forensic populations given the positive effects reported in most studies and the ability to tailor interventions to patient-specific needs [17]. However, this review also pointed out that the advantages of eHealth and mHealth heavily depend on integration into treatment and fit with the needs and preferences of patients and therapists.

Considering the needs and preferences of intended users is one of the main prerequisites to bridge the gap between promising results of eHealth and mHealth studies on the one hand and actual deployment of these interventions in (forensic) mental health care on the other [18]. In addition to specific characteristics of patients and therapists, technological aspects, internal implementation climate, and external policy incentives contribute to the uptake of new interventions [19,20]. These factors share many similarities with the levels of an often-used implementation model designed to understand and inform the process of change in health care [21]. According to this model, barriers and incentives to change can be categorized into 6 different levels: innovation (eg, feasibility and accessibility), individual professional (eg, awareness, knowledge, and behavioral routines), individual patient (eg, skills, attitude, and compliance), social context (eg, opinion of colleagues and collaboration), organizational context (eg, organization of care processes, staff, and resources), and economic and political context (eg, financial arrangements and policies). In a recent systematic review, 3 levels of barriers and success factors concerning the implementation of eHealth services were identified: technical factors (eg, usability, security, and support), individual factors (eg, cognition, motivation, and trust), and environmental and organizational factors (eg, financing, proof of effectiveness, and fit into organizational structures) [22]. This seems to be a more parsimonious model as individual levels are merged and internal and external organizational barriers are combined.

### Aim of This Study

Given the scarcity of information on the deployment of sensor-based mHealth interventions in (forensic) mental health care, we aimed to provide more insight into the perspectives of both forensic outpatients (study 1) and therapists (study 2) regarding the use and implementation of a new sensor-based mHealth intervention, the Sense-IT biocueing app, in aggression regulation therapy (ART). More specifically, we focused on facilitators of and barriers to implementation, as identified by previous models. As we expected the feedback of forensic outpatients to be mainly centered on the technical or innovation level and the individual patient level, we included forensic therapists to provide us with information on all levels of the implementation model.

## Methods

### Study 1

#### Design

The perspectives of forensic outpatients on the use of the Sense-IT biocueing app were explored using qualitative semistructured interviews. In addition, usability was determined using a quantitative usability score. Data were collected at the postmeasurement assessment (T1) within a larger quasi-experimental study in which patients used the Sense-IT app for 4 weeks. More specific information on the design of this study can be found in another paper [23].

#### Recruitment

Forensic outpatients who received ART at Inforsa, a forensic mental health care organization in Amsterdam, the Netherlands, were recruited for participation between January 2020 and March 2022. First, patients were screened for eligibility by a research assistant, consulting the patients’ therapist. Patients were eligible for participation if they lacked anger management skills, were assigned to individual ART, had a basic understanding of mobile apps, and were aged ≥16 years. Patients were excluded if they had acute manic or psychotic symptoms, a high risk of suicide, severe physical conditions requiring immediate intervention, or insufficient understanding of the Dutch language. If a patient turned out to be eligible and interested in the research project, study participation was offered in a face-to-face appointment in which a brief oral description and full written information were provided.

#### Procedure

After screening and informing the patients about the research project, 25 patients were eligible and willing to participate. All patients participated in a baseline measurement (T0) in which demographic characteristics, attitudes toward new technologies, and perceived proficiency in using new technologies were assessed. Next, they were provided with the Sense-IT app for 4 weeks, in which they received biocueing for 2 weeks. Using the photoplethysmography sensor of a smartwatch, this app compares the users’ current heart rate (HR) to their individual mean HR at rest, calculating a level between −3 and 5 using the SD of the baseline measurement. The app provides a visual display of the real-time HR level on the smartwatch and smartphone and notifies the users when their HR exceeds a predefined level using notifying vibrations and behavioral support messages. Screenshots of the Sense-IT app can be found in Multimedia Appendix 1. The System Usability Scale (SUS) and the semistructured interviews were assessed after 4 weeks, at the postmeasurement assessment (T1). The SUS is a short, widely used Likert-scale questionnaire for quick and reliable assessment of usability [24], yielding an overall score between 0 and 100. According to recent research, a product with scores >70 is acceptable, whereas better products score in the high 70s to upper 80s and superior products score >90 [25]. The semistructured interviews consisted of 27 questions investigating attitudes toward new technology, usability, and efficacy. For this study, we focused on 5 open questions regarding the advantages and disadvantages of the Sense-IT biocueing app, specific situations in which the app was assessed as pleasant or useful, and suggestions regarding future use.

#### Ethics Approval

This study was part of a larger study approved by the Medical Ethical Committee (NL63911.029.17) and registered in the Netherlands Trial Register (NL8206). Patients were carefully informed about the anonymous use of data, the voluntary nature of the study, and the absence of any negative consequences in case of refusal or early termination. All patients provided written informed consent before participation. Participating patients received a gift card of €10 (US $10.81) and an additional €10 (US $10.81) at T1 when at least 75% of the repeated experience sampling questions (not further reported here) were answered.

#### Data Analysis

We organized the qualitative data of the outpatients using Microsoft Excel (Microsoft Corp). For categorical responses, cumulative frequencies were calculated using SPSS (version 27; IBM Corp). For open responses, content analysis was used. First, textual responses were inspected by the first author (JFtH) to gain familiarity with the data and establish a coding scheme. Next, the first and second authors (JFtH and LMS) independently coded the responses into predefined categories. Discrepancies were discussed between both authors until a consensus was reached. Discussion was also used to refine the categories and code descriptions to increase the interpretation of the codes. Quotes were jointly selected by both authors considering their informative value and ability to illustrate the category. Finally, selected quotes were translated from Dutch into English by JFtH and LMS.

### Study 2

#### Design

The perspectives of forensic therapists on the implementation of the Sense-IT biocueing app were explored using focus groups. This study was designed to investigate the added value of the app from a therapist’s point of view, gain a better understanding of the perceived facilitators of and barriers to implementation, and collect suggestions for implementation.

#### Recruitment

Forensic therapists working within outpatient teams at Inforsa were recruited by email in February 2022. Therapists were eligible and invited to participate if they were trained in ART. No other inclusion or exclusion criteria were applied.

#### Procedure

In total, 2 focus groups were conducted in February 2022 and March 2022. Both focus groups were scheduled within the existing structure of team meetings to avoid time burdens and added workload, thereby enhancing the chances of participation. In line with this structure (and also relevant from a content perspective), separate focus groups were organized for therapists working with young adults and therapists working with adults. Owing to COVID-19 regulations, some therapists participated on site, and others participated on the web. The planned group size was 6 to 8 participants to allow for optimal interaction between them [26]. The focus groups were conducted by a moderator (JFtH) and an assistant (LMS). The moderator was a licensed health care psychologist with extensive training in interviewing skills and techniques. The moderator and assistant were both affiliated with Inforsa as scientist practitioners. They were not employed as therapists in the teams at the time the focus groups were held.

At the start of the focus groups, a brief overview of the Sense-IT project was presented, including background information, screenshots of the app, and some qualitative results of earlier studies. After that, therapists completed a short form assessing demographic characteristics, attitude toward both mHealth in mental health care and new technologies, and perceived proficiency in using new technologies. In both focus groups, the same questioning route was used (Multimedia Appendix 2). The moderator structured the discussion to cover key themes but was also responsive to issues emerging in the focus groups. The questioning route was developed by the researchers and adapted using feedback from important stakeholders (researchers, therapists, and policy makers). To assess the added value, specific questions were used to discuss the impact on the therapist, patient, and treatment level. Questions to assess the perceived barriers to and facilitators of implementing the Sense-IT biocueing app were aligned with the 2 aforementioned implementation models [21,22]. The focus groups lasted approximately 1 hour.

#### Ethical Considerations

For this study among therapists, no ethical board review statement was applicable given the embedding in regular care routines and the low burden of participation. Therapists were informed that participation was voluntary, that information would be processed anonymously, and that the data would be used for research and policy purposes as well as for enhancement of the implementation process of the Sense-IT biocueing app. Before participation, therapists received an informative email and an informed consent form. All participants provided written informed consent. Participating therapists did not receive any financial reimbursements.

#### Data Analysis

The focus groups were video- and audiotaped, transcribed in full by LMS, and analyzed by JFtH and LMS using content analysis. We used a combination of open and axial coding in a process of constant comparison [27]. First, JFtH and LMS both read the verbatim transcriptions to gain familiarity with the data. Next, JFtH established an initial coding scheme to categorize the responses into the predefined levels. JFtH and LMS independently categorized and open-coded the responses using MAXQDA 2022 (VERBI GmbH) [28]. The assigned categories (levels) and codes (subthemes) were compared using the merge function in MAXQDA. JFtH and LMS first reached intercoder agreement on levels by discussing the disagreements in categorization until a consensus was reached. Next, the researchers discussed the codes, resulting in joint axial coding and refinement of the subthemes. After that, the subthemes were jointly categorized into main themes, thereby connecting individual subthemes with the predefined levels. Finally, both researchers reached a consensus on the facilitator and barrier annotations of the codes. The refined coding scheme and completed analysis were verified by the last author (TMvdP). Discrepancies were discussed by JFtH, LMS, and TMvdP until a consensus was reached. Finally, important themes and subthemes were identified, and informative quotations that could enhance the interpretation of the results were selected and translated from Dutch into English by JFtH and LMS.

## Results

### Study 1

#### Descriptive Statistics

In total, 21 forensic outpatients (n=19, 90% male and n=2, 10% female) filled out the SUS and participated in the interview at T1. Most participants (14/21, 67%) received mandatory treatment as a part of a conditional sentence. The main psychiatric disorders most frequently classified according to Diagnostic and Statistical Manual of Mental Disorders, Fifth Edition, criteria were disruptive disorders (9/21, 43%) and personality disorders (8/21, 38%). Some participants were diagnosed with intellectual disability or scored below the cutoff on a screener for mild intellectual disability (8/21, 38%). At posttest measurement, forensic outpatients evaluated the usability of the Sense-IT biocueing app as acceptable (mean 73.13, SD 13.35). System usability was not significantly correlated with age, attitude toward new technology, or perceived proficiency in using new technologies. Notably, all patients returned the borrowed materials except for 10% (2/21), who reported that they had lost their smartwatch because of robbery. All descriptive characteristics are summarized in Table 1.

**Table 1:** Descriptive characteristics of forensic outpatients (study 1; N=21).

Variable		Values
Age (years), mean (SD)		29.76 (10.60)
Male participants, n (%)		19 (90)
**Cultural background, n (%)**		
	Western	7 (33)
	Non-Western	7 (33)
	Mixed	7 (33)
**Educational background, n (%)**		
	None	1 (5)
	Primary education	3 (14)
	Junior secondary education	12 (57)
	Senior secondary education	5 (24)
Attitude toward new technologies, mean (SD)^a^		4.33 (0.86)
Perceived proficiency in using new technologies, mean (SD)^b^		8.00 (1.05)

^a^Measured on a 5-point Likert scale, with 1 indicating a (very) negative attitude and 5 indicating a (very) positive attitude.

^b^Graded on a 10-point scale, with 1 indicating no proficiency and 10 indicating excellent proficiency.

#### Results

Forensic outpatients identified a wide range of advantages and disadvantages of the Sense-IT biocueing app. Responses mentioned more than once that could be grouped into categories are presented in Table 2.

Clarity and simplicity of the app as well as support in interoceptive and emotional awareness were most frequently reported as advantages. Most patients (17/21, 81%) reported no difficulty in understanding how to use the app, and some (7/21, 33%) explicitly indicated that the app was clear and well organized. Furthermore, patients indicated that the app helped them become more aware of physical tension and that the questions in the app assisted them to reflect on their emotions and behavior during the day. A participant reported the following:

[The app helped me] to reflect on how things were going; I never really did that, but now I was aware whether I had a good day or a not so good day.

Participant 10

Connectivity issues, notification issues, and perceived inaccuracy were mentioned the most as disadvantages. Regarding connectivity issues, patients reported disturbance via interruptions in the Bluetooth connection, for example, when the distance between the smartphone and smartwatch was too large. As a potential solution, a patient suggested running the app stand-alone on the smartwatch itself so no Bluetooth connection would be needed. Furthermore, a substantial number of participants (8/21, 38%) reported that they received too many notifications or notifications that they perceived as either too soon or too late. In addition to the ability to adjust the (maximum) number of notifications, patients suggested sending notifications only when a higher HR was registered over a longer time. A participant recommended the following:

Add a button to put the app on pause, as a time-out, when you get irritated by the number of notifications, or when you already know [that you are tense].

Participant 21

Related to this, several patients (6/21, 29%) indicated that they would have preferred to customize the settings of the app themselves; their ability to do so was restricted in this study. For example, they would have liked to be able to adjust the number of notifications as well as the frequency and content of the daily questions according to their preferences. Furthermore, patients questioned the accuracy of the feedback provided by the app. Patients mentioned both elevations in HR when they did not subjectively experience stress and subjectively experiencing stress without detected elevations in HR. Patients reported that their activity profiles were not always recognized correctly by the app. Furthermore, patients reported missing specific design features such as the use of colors (eg, red color to signal high tension), graphical overviews, and more variety in watch faces (ranging from a very clear watch face with the actual HR to a watch face that is less easy to interpret for others). Other frequently mentioned disadvantages were the use of a study-provided smartphone and the limited battery life of the smartwatch.

Furthermore, we assessed in which specific situations the app was described as (not) pleasant or useful. Half (11/21, 52%) of the patients reported no specific situations or no situations at all in which they perceived the app as pleasant or useful. In retrospect, the app was perceived as most useful (mentioned by 7/21, 33% of the patients) in or shortly after discussions, confrontations, and other situations with a lot of tension to support awareness and emotion regulation. Related to this, the app was judged as not pleasant or useful in relaxed settings in which notifications perceived as inaccurate were reported as disturbing (5/21, 24% of the patients), during exercise or other physical activities (6/21, 29% of the patients), or when patients already felt too stressed or tired (3/21, 14% of the patients). A participant summarized this as follows:

In places where you have a lot of tension: it is good to put it [the smartwatch] on just then, and not in situations when you are calm.

Participant 25

**Table 2:** Advantages and disadvantages of the Sense-IT biocueing app and the frequency with which each code was identified in the responses of the forensic outpatients (study 1; N=21).

Code			Code frequency, n (%)		Definition
**Advantages**					
	Rationale	4 (19)		The idea or rationale behind the app; its functionality	
	Simplicity	7 (33)		The clarity and simplicity of the app and its functions	
	Awareness	8 (38)		The helpfulness of the app to increase both interoceptive and emotional awareness	
	Behavioral support	2 (10)		The helpfulness of the behavioral support messages	
**Disadvantages**					
	Perceived inaccuracy	7 (33)		The perceived inaccuracy of the HR^a^ measurements or the recognized activity profiles or the perceived limitations of the app to detect subjectively experienced stress	
	Notification issues	8 (38)		Problems related to the amount (too many) or the timing (too soon or too late) of the notifications received	
	Connectivity issues	8 (38)		Problems related to instability of the connection between smartwatch and smartphone	
	Use of a study-provided phone	5 (24)		Problems related to the use of the app on a study-provided smartphone	
	Limited adaptive functionalities	6 (29)		Limitations in the ability to personalize settings during the study	
	Design-related issues	6 (29)		Problems related to personal design-related preferences	
	Other software issues	4 (19)		Other problems related to the functions of the app	
	Limited battery life	4 (19)		Limitations in the battery life of the smartwatch	
	Other hardware issues	4 (19)		Other problems related to the smartwatch	

^a^HR: heart rate.

### Study 2

#### Descriptive Statistics

In total, 21 forensic therapists were invited to participate in the focus groups. A total of 24% (5/21) of the therapists preannounced that they were unable to participate for practical reasons. One new therapist, who did not receive the invitation, indicated willingness to participate and joined one of the focus groups. Eventually, another 10% (2/21) of the therapists did not participate. In total, 2 focus groups were conducted: one for therapists working with young adult patients (focus group 1; 6/15, 40%) and one for therapists working with adult patients (focus group 2; 9/15, 60%). In Table 3, descriptive characteristics of the participants are presented.

**Table 3:** Descriptive characteristics of the forensic therapists (study 2; N=15).

Variable		Focus group 1 (n=6)	Focus group 2 (n=9)
Age (years), mean (SD)		35.00 (5.55)	35.11 (7.99)
Female participants, n (%)		5 (83)	8 (89)
**Position, n (%)**			
	Master psychologist or pedagogue	4 (67)	3 (33)
	Health care psychologist (in training)	0 (0)	3 (33)
	Clinical psychologist (in training)	1 (17)	3 (33)
	Systemic therapist	1 (17)	0 (0)
**Work experience (years), n (%)**			
	<5	1 (17)	3 (33)
	5-10	2 (33)	1 (11)
	>10	3 (50)	5 (56)
**Work experience in forensic psychiatry (years), n (%)**			
	<5	4 (67)	9 (100)
	5-10	1 (17)	0 (0)
	>10	1 (17)	0 (0)
Attitude toward mHealth^a^ in mental health care, mean (SD)^b^		4.67 (0.52)	3.89 (0.93)
Attitude toward new technologies, mean (SD)^b^		4.50 (0.55)	3.22 (1.30)
Perceived proficiency in using new technologies, mean (SD)^c^		7.75 (1.41)	6.72 (2.68)

^a^mHealth: mobile health.

^b^Measured on a 5-point Likert scale, with 1 indicating a (very) negative attitude and 5 indicating a (very) positive attitude.

^c^Graded on a 10-point scale, with 1 indicating no proficiency and 10 indicating excellent proficiency.

#### Results

##### Added Value

**Figure 1:**
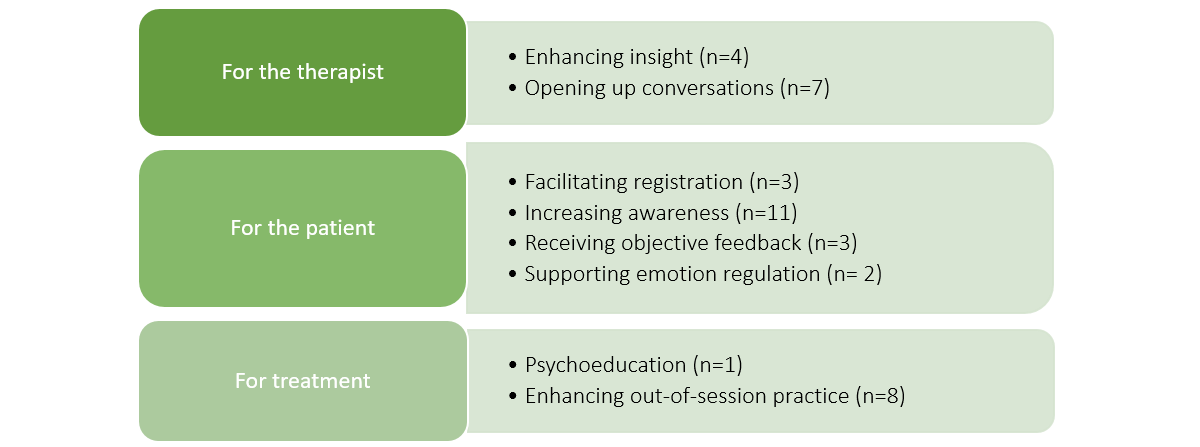
Patterns of meaningful responses (with code frequency) regarding the added value of the Sense-IT biocueing app according to forensic therapists (study 2).

In the first step of our analysis, we retrieved 39 codes from the 2 focus groups related to the added value of the Sense-IT biocueing app from the therapists’ perspective. These codes were categorized into themes and linked to the predefined levels in the questioning route. An overview of the results of this part of the coding process is shown in Figure 1.

At the therapist level, using the app to open up conversations was mentioned most frequently. A therapist noted the following:

I believe it’s great to be able to discuss with patients, who sometimes already forgot what they did yesterday...to zoom in on specific moments, to start talking about it.

Therapist 1, focus group 1

At the patient level, increasing interoceptive and emotional awareness was mentioned the most. Therapists supposed that the app could help patients learn and experience how their body and mental state are connected and that reminders could help them be aware of the signals the body gives in everyday life. Furthermore, a therapist stressed the importance of these basal skills in the first stages of treatment:

I realize that body awareness precedes all those cognitive things.

Therapist 4, focus group 2

At the level of treatment itself, therapists most frequently mentioned the impact on out-of-session practice, thereby increasing the transfer to everyday life. Therapists noted that wearing the smartwatch and using the app might also function as reminders for patients that they are in a process of learning to control their aggressive responses. They reported the following:

I suppose it can also help patients to be engaged in their treatment outside the therapy session. So it’s easier to generalize what you are doing [in therapy].

Therapist 4, focus group 2

The therapy does not end in the room, but continues in daily life. The watch could also be a reminder for patients.

Therapist 1, focus group 2

##### Facilitators and Barriers

###### Overview

In the second step of our analysis, 92 codes were retrieved on facilitators of and barriers to the implementation of the Sense-IT biocueing app in current treatment. The coded subthemes as well as the later defined themes were linked to the previously described levels [21,22]. An overview of the results of this coding process is presented in Table 4.

**Table 4:** Results of the coding process of facilitators of and barriers to implementation of the Sense-IT biocueing app in current treatment, as mentioned by forensic therapists (study 2)^a^.

Levels by Schreiweis et al [22], levels by Grol and Wensing [21], themes, and subthemes					Focus group 1					Focus group 2		
					Facilitators		Barriers		Facilitators			Barriers
**Technical**												
	**Innovation (n=13 codes)**											
		**Usability**										
			(Limited) ease of use	✓^b^		✓					✓	
			Problems with materials			✓						
			(Limited) simplicity	✓		✓		✓				
		**Perceived accuracy**										
			Limited perceived accuracy								✓	
**Individual**												
	**Therapist (n=28 codes)**											
		**Workload**										
			Added workload			✓					✓	
		**Integration into treatment**										
			Integration into treatment	✓				✓				
		**Knowledge and skills**										
			(Limited) technological skills	✓		✓					✓	
			Familiarity with the app	✓				✓				
	**Patient (n=33 codes)**											
		**Motivation**										
			Lack of problem insight			✓						
			(Limited) openness to feedback	✓		✓					✓	
			(Limited) motivation	✓		✓		✓			✓	
		**Specific problems**										
			Feeling controlled			✓					✓	
			Specific psychiatric characteristics			✓		✓			✓	
		**Knowledge and skills**										
			(Limited) technological skills	✓		✓		✓			✓	
			Cognitive problems			✓					✓	
			(Not) taking care of materials	✓		✓					✓	
			Practical issues			✓						
**Environmental organizational**												
	**Social context (n=3 codes)**											
		**Expertise**										
			(Limited) expertise in team	✓		✓		✓			✓	
	**Organizational context (n=12 codes)**											
		**Time**										
			Providing sufficient time	✓								
		**Materials**										
			(Lack of) clear agreements	✓		✓		✓				
			(Problems in) providing materials	✓		✓		✓				
	**Political and economic factors (n=3 codes)**											
		**Knowledge and insight**										
			Knowledge of effectiveness					✓				
			(Limited) insight into costs and benefits	✓		✓						

^a^Therapists working with young adults participated in focus group 1, and therapists working with adults participated in focus group 2.

^b^✓: indicates whether a theme was mentioned in the focus group and discussed as a facilitator and/or barrier.

###### Technical or Innovation Level

*Usability* was identified as an important issue for implementation. The limited ease of use of the app on a research-owned smartphone was discussed as a barrier in both focus groups. The use of the app on the patients’ own smartphones was explicitly mentioned as a facilitator by the therapists in the young adult group. They explained that these patients almost never lose sight of their own phones. Therapists in this group also reported potential problems with the materials, such as loss or damage to the devices or charging cables. Furthermore, the simplicity of the rationale of the app—delivering concrete, real-time physiological feedback—was mentioned as a facilitator. Both groups discussed whether the design of the app was easy enough for patients with intellectual disabilities. However, the therapists did not reach a conclusion regarding the ease of use for this group, indicating that the accessibility of the app for patients with intellectual disabilities might need further investigation.

*Perceived accuracy* of the app was also discussed as an essential factor. In the focus group of therapists working with adults, limitations in the perceived accuracy of the feedback were mentioned as barriers. One of the therapists mentioned that receiving too many notifications or notifications perceived as inaccurate may reduce the likelihood of continued use of the app:

The app should provide notifications at the right moments...so that patients continue to take the app seriously.

Therapist 1, focus group 2

###### Individual Level—Therapist

*Workload* was expected to be increased by implementing the app and, therefore, was identified as a barrier in both focus groups. Therapists expressed some concerns regarding the time and continuous attention needed for implementation of the app in their current routines. Therapists noted that there are already a lot of other topics to discuss during a therapy session. To maintain the use of a new intervention by patients, therapists reported that they would need to address this frequently during sessions. A therapist explained that the investment of time would be large in the beginning but probably less over time when therapists can take a more distant position.

*Integration into treatment* was mentioned as a facilitator of implementation in both focus groups. A therapist explained that it would be valuable to view a graphical overview of the patients’ physiological values and notes over the last week, before or at the start of a therapy session, and discuss this with the patient. In the group of therapists of the adult population, integration of the app in the process of creating a personal monitoring plan was suggested. Furthermore, therapists mentioned that the use of the app should be an integral part of their treatment plan. These therapists indicated a need for criteria and guidelines helping them indicate whether the intervention would be beneficial for a particular patient.

*Knowledge and skills* of therapists were also identified as important factors. Limited proficiency in using technological interventions was mentioned as a barrier in both groups. In the focus group of therapists of young adults, the facilitating impact of technological skills and a positive attitude toward new technologies were also mentioned. In both groups, therapists expressed interest in and supported the relevance of becoming more familiar with the app by using it themselves. A therapist explained this as follows:

I can imagine that it helps if you can say, from your own perspective, that using the app can be a bit irritating at times. It might be useful to relate from your own experience.

Therapist 6, focus group 2

###### Individual Level—Patient

Factors related to *motivation* were discussed in both groups. A lack of problem insight or disagreement on the aggressive nature of specific behaviors was identified as a barrier to implementation in one of the focus groups. Both focus groups mentioned a certain amount of motivation (or at least ambivalence) to change as a facilitator, and the lack thereof was mentioned as a barrier. In one of the focus groups, using the app to increase motivation and problem insight was also discussed. Therapists also mentioned the ability to be open and receptive to feedback, which could be confronting or annoying, as an important issue. A therapist explained this as follows:

You need to have the courage to start looking at yourself.

Therapist 3, focus group 1

*Specific problems* may also complicate implementation. In both groups, the feeling of being controlled by others was seen as a contraindication for the use of the app. In addition to delusional and other psychotic disorders, disorders that involve an excessive focus on physiological sensations, for instance, hypochondria, were also mentioned as barriers. Furthermore, therapists perceived the app as most useful for patients with reactive aggression and not indicated for patients with predominantly instrumental or proactive aggression.

*Knowledge and skills* of the patients were also discussed as important issues. Proficiency in using technological interventions was mentioned as a facilitator in both groups, and the lack thereof was mentioned as a barrier. Cognitive problems or intellectual disabilities were identified as barriers in both groups, although the app was also perceived as particularly helpful for this group of patients as they often lacked insight into the (physiological) signals that precede aggressive behavior. Therapists in both groups expressed some concerns that patients would lose or sell the devices. Therefore, they discussed the ability to take adequate care of the devices and a certain degree of responsibility for others’ belongings as prerequisites for borrowing materials. In the young adult group, practical issues related to unstable circumstances such as homelessness were also mentioned as barriers.

###### Environmental and Organizational Level—Social Context

*Expertise* within the team was seen as an important facilitator (and the lack thereof was seen as a barrier) for sustainable use of the app in both groups. Therapists discussed the risk of dilution in case no one within the therapists’ team felt ownership of the app. A therapist suggested a special interest group, whereas another proposed appointing one therapist per team as an expert in using the app. This last suggestion was supported by the members of the other focus group. A therapist stressed the importance of expertise by reporting the following:

If all therapists just occasionally use the watch, then all will lack expertise.

Therapist 3, focus group 1

###### Environmental and Organizational Level—Organizational Context

*Time* to become familiar with the app was explicitly mentioned as a facilitator in one of the focus groups. This was related to the expectation of added workload associated with the addition of the app to current therapy. A therapist reported the following:

If spending a lot of time on the app at the start is facilitated at the organizational level, then it can be done.

Therapist 4, focus group 1

*Materials* were also discussed in both teams. Therapists working with young adults stressed the facilitating impact of having the devices directly available in their offices. They explained that this is not only helpful to introduce the app to their patients but also to initiate using the app when the moment is right. Therapists made it clear that they had no desire to fill in (extensive) application forms; they would like the organization to provide them with materials in an uncomplicated way. Furthermore, both groups discussed the lack of clear agreement on the use of devices by patients as barriers. Therapists in both groups explained that it would be helpful if the organization provided a kind of contract in which agreements on loss, theft, and liability were included.

###### Environmental and Organizational Level—Political Context

*Knowledge and insight* were briefly mentioned in both groups. Knowledge of effectiveness was mentioned as a facilitator in one group, whereas insight into costs and benefits was discussed as a facilitator and barrier in the other. These therapists explained that more proof of the effectiveness of the app and more information on the costs compared with treatment as usual would increase their willingness to use the app and, thereby, the likelihood of successful implementation.

####### Other Suggestions

The therapists provided us with many other valuable suggestions regarding the use and implementation of the Sense-IT biocueing app, which will be used to inform the future process. In the context of this paper, 2 topics were highlighted. First, several therapists (4/15, 27%) recommended the use of the app in the first phases of treatment to increase awareness of physiological signals that precede aggressive behavior. Other therapists (2/15, 13%) suggested the use of the app in later phases of treatment; one therapist reported focusing on motivation first, whereas another suggested using the app during therapy sessions to evaluate different emotion regulation strategies. Second, therapists working with young adults explored opportunities for a more systemic approach. Therapists mentioned that the use of the app could probably increase awareness among system members as well and might help enable system members to adequately support the patient in moments when physiological tension is elevated.

## Discussion

### Principal Findings

The aim of this study was to provide insight into the perspectives of both forensic psychiatric outpatients and therapists on the use and implementation of a new sensor-based mHealth intervention for ART. More specifically, we aimed to obtain a more in-depth understanding of the facilitators of and barriers to implementation, which could be used as guideposts for future research and clinical practice. Findings from both studies indicate an overall positive attitude toward the addition of a biocueing intervention in forensic therapy, with increased interoceptive and emotional awareness as the most frequently and commonly mentioned advantage. The main barriers at the *technical or innovation level* mentioned by both patients and therapists were several usability issues (ie, limitations in the ease of use, such as connectivity and notification issues; limitations in the ability to personalize settings; and problems related to the devices, such as a limited battery life of the smartwatch) as well as limitations in the perceived accuracy of the feedback. For the *individual therapist*, added workload and limited technological skills were perceived as barriers, whereas integration into treatment and familiarity with the app were mentioned as facilitators. For the *individual patient,* motivation and knowledge and skills were discussed as both barriers and facilitators of implementation success. Specific psychiatric problems (ie, paranoia and hypochondria) were identified as barriers.

At the *environmental and organizational level*, sufficient expertise within the therapists’ team was seen as a prerequisite for implementation. For organizations, providing time to become familiar with the innovation and providing (clear agreement on the use of) materials were identified as important facilitators. More knowledge of and insight into the effectiveness and cost-effectiveness of the intervention were identified as political or economic factors influencing uptake by therapists.

The findings regarding the perceived advantages of this sensor-based biocueing app resonate with the results of previous mHealth studies. Increased awareness (mentioned by both patients and therapists) and the ability to open up conversations by zooming in on specific situations (mentioned by therapists) align with previously described benefits of mHealth [22,29]. Extending the reach of therapy through out-of-session practice (mentioned by therapists), thereby enhancing treatment adherence and facilitating the treatment process, has also been identified as one of the unique opportunities afforded by mobile apps [30]. This is particularly important as motivation, problem insight, and treatment adherence are typically low in forensic populations. The potential of eHealth and mHealth in forensic populations [17] seems also supported by the positive attitude toward new technologies and the perceived proficiency in using these technologies of the participating forensic outpatients and therapists working with young adults.

Barriers and facilitators identified in the focus groups were linked to the 3 factors associated with eHealth implementation [22] and the levels of a more general implementation model [21].

The disadvantages mentioned by the patients corresponded, to a large extent, to the barriers at the *technical or innovation level* discussed by the therapists. Although the overall usability of this particular biocueing app turned out to be acceptable, frequently mentioned problems in using a biocueing intervention (eg, connectivity and notification issues and limitations in smartwatch battery life) need further attention as end users tend to stop using a health app when their preferences and goals are not met [31]. Usability is also expected to be enhanced when patients can use the intervention in line with their personal preferences and on their own smartphones outside a strict research context. Self-adjustment of the settings, as well as the suggested pause button, could also help reduce irritation and disappointment caused by the number of notifications and perceived inaccurate feedback. Although irritation might be partly explained by limitations in frustration tolerance among forensic outpatients, feelings of disappointment might also originate from (very) high expectations of what the app should deliver. Biocueing interventions can identify substantial increases in arousal by measuring HR, but they are unable to provide a unique and specific recognition of subjectively experienced stress or specific emotion categories [32]. As suggested in recent research in which patients reported similar feedback [33,34], a more detailed explanation of biocueing might help create more realistic expectations.

The barriers identified at the *individual therapist level*—added workload and limited technological skills—have been identified in earlier reviews [22,30]. Integration of the eHealth or mHealth intervention into regular treatment and familiarity with the system have also been listed as 2 of the most important facilitators of implementation [22,30]. These factors are also related to the level of adoption of these interventions, as described in the Levels of Adoption of eMental Health model [35]. According to this model, the adoption levels of most of the participating therapists could be considered as minimal use (level 2) or passive use (level 3); a smaller proportion would fit into the category of active users (level 4). In this sample, the highest scores on attitude toward new technology and the use of mHealth as well as on perceived proficiency in using new technologies were found among the therapists working with young adults, which might parallel the usually higher efficacy in using new technological interventions found among young people [36].

At the *individual patient level*, therapists indicated a certain amount of motivation and problem insight as a prerequisite to benefit from the biocueing intervention. However, other therapists also saw potential to use the app as a means of increasing problem insight. This difference might be related to the 2 components of this biocueing intervention: increasing interoceptive awareness and delivering just-in-time behavioral support. Although the second component might require some problem awareness and receptivity to feedback, as demonstrated in another biocueing study [37], this might not be necessary to benefit from the first component. Regarding knowledge and skills, patients rated their proficiency in using new technologies as high and the simplicity of the app as an advantage. However, therapists discussed whether the current version of the biocueing app was accessible enough for patients with intellectual disabilities. Therefore, accessibility and potential adaptations for this particular group could be further assessed in future research [38-40]. Concerns of forensic therapists about young adults’ ability to take care of the devices should be taken into account, although in this study, only 2 devices were not returned by patients. Furthermore, lack of trust and the sense of feeling controlled by others were identified as contraindications. Although these feelings may occur in the context of a psychiatric disorder, they might also originate from legitimate concerns about becoming an object of surveillance and persuasion as commercial apps often own the right to share and sell collected personal health data [41] and health-related suggestions can start to feel as an invasion of personal space [42]. In our study, we accounted for these issues by ensuring data safety using local storage and stressing the voluntary nature of participation.

Finally, at the *environmental and organizational level*, most barriers were addressed in the organizational context. The need expressed by therapists to be provided with sufficient time and material by their management was also identified as an important implementation factor in the literature [22,30]. To avoid ambiguities that might interfere with the therapeutic process, therapists also recommended clear agreements on the use of smartwatches (and smartphones, if applicable) by patients. Also mentioned for successful implementation were the importance of expertise within the therapists’ team as well as the need for more information on effectiveness and cost-effectiveness, which has been reported previously as essential for providing a solid embedding of new interventions in the health care system [43].

### Strengths and Limitations

This study has several strengths. First, we were able to assess the experiences and perspectives of forensic outpatients, who are often hard to engage in clinical research. Most participants seemed to enjoy delivering feedback on how to improve this new sensor-based mHealth intervention. Second, we combined the information of forensic outpatients with the input of forensic therapists to obtain a more complete overview of the barriers and facilitators associated with implementation. To do so, we combined a more general implementation model with a more specific eHealth implementation model. Finally, this study was well embedded in daily clinical practice, thereby enhancing the ecological validity and translation of these research results into real-world situations.

Our study also had several limitations. In both studies, attitudes toward new technologies (and mHealth) as well as perceived proficiency in using those technologies were assessed using self-developed Likert-scale questions. The use of recently developed and validated scales such as the eMental Health Adoption Readiness scale [44] could have contributed to a more accurate assessment of this highly relevant aspect of eHealth and mHealth implementation. In the study of forensic outpatients, several factors related to the research design (such as the use of a research-owned smartphone and the restricted ability to customize settings) impeded usability, which might have negatively influenced the overall experience with the biocueing app. Furthermore, as both studies were conducted within 1 forensic outpatient organization, some barriers and facilitators might not apply to other organizations. Finally, as the moderator has been working as a scientist practitioner at the organization for the past years, this might have created some positive bias in the responses.

### Implications for Future Research and Practice

In addition to suggestions for further improvement of this particular biocueing app and other issues requiring attention (ie, perceived accuracy of the app, accessibility for patients with intellectual disabilities, and ethical concerns regarding surveillance and persuasion), this study provided valuable information to guide the implementation of sensor-based mHealth interventions in (forensic) mental health care. As the implementation of eHealth and mHealth largely depends on the providers of these interventions [45], the individual and team differences in adoption readiness should be taken into account [35,46]. Considering the adoption levels of most of the therapists in this study, implementation should first be focused on enhancing integration into daily routines and, after that, on increasing familiarity and affinity with the intervention. Active users could be given a role as experts within the teams, supporting their colleagues to explore the possibilities of the new intervention.

Therapists and patients provided several suggestions for using a biocueing app in clinical practice. Although these suggestions specifically apply to the studied intervention, they might also be informative for other researchers developing and implementing similar sensor-based mHealth interventions in (forensic) mental health care. According to the therapists, the addition of a biocueing intervention would be most useful in the first phases of treatment to increase interoceptive awareness. When patients display a certain amount of motivation to change and receptivity to feedback, they might also benefit from the just-in-time behavioral support delivered by the biocueing app. As this involves a reminder to use coping skills to reduce stress, it is necessary that the therapist has already discussed emotion regulation strategies with the patient and that the behavioral support message is prepared in collaboration. Patients indicated that the app was most helpful in difficult situations, and it was perceived as disturbing in relaxed situations, during exercise, or when they already felt too stressed or tired. This emphasizes the need to further adapt these new interventions to deliver mental health support at precisely those moments, when they are most likely to be effective [47]. Therefore, biocueing interventions align well with the already initiated shift toward personalizing treatment in mental health care [48,49]. In the development and implementation of these interventions, it is important to aim for an optimal fit between user experience, effectiveness, privacy and data safety, and data integration into treatment routines [31].

### Conclusions

Forensic outpatients and therapists demonstrated a positive attitude toward the addition of a wearable sensor–based mHealth intervention, the Sense-IT biocueing app, to ART. Increased interoceptive and emotional awareness were mentioned as advantages by both patients and therapists. However, to maximize the potential of these interventions, several important barriers and facilitators should be addressed. Forensic outpatients mainly reported technical or innovation-related barriers, whereas therapists provided us with a more in-depth understanding of barriers and facilitators at the individual and organizational levels. A substantial part of the technical or innovation-related barriers is related to the developmental stage of the app and its use in a research context, and therefore, quite easy to address. Furthermore, although some individual patient barriers apply specifically to forensic patients, most factors should also be carefully considered in other populations with emotion regulation difficulties. At the individual therapist and organizational level, providing time and materials supporting integration into daily routines and enhancing affinity with the new intervention were identified as important facilitators of implementation and, therefore, are highly recommended. In the future implementation process, individual and team differences in readiness for adoption of mHealth should be considered, assigning a central role to active users as experts within the teams. Finally, as further development of biocueing interventions is expected, new and personalized app possibilities might be discovered and investigated at the individual patient level, aligning with the trend of personalizing treatment interventions in mental health care.

## Appendix

Multimedia Appendix 1 Screenshots of the Sense-IT biocueing app (version 2.57): the main screen, measurement screen, notes screen, and one of the watch faces.

Multimedia Appendix 2 Questioning route.

## Abbreviations

ART: aggression regulation therapy
HR: heart rate
mHealth: mobile health
SUS: System Usability Scale

## Data Availability

The data sets generated and analyzed during this study are available from the corresponding author upon reasonable request.
