# Supplementary figures and images for: Forensic Psychiatric Outpatients’ and Therapists’ Perspectives on a Wearable Biocueing App (Sense-IT) as an Addition to Aggression Regulation Therapy: Qualitative Focus Group and Interview Study
Source: JMIR Form Res. 2023 Feb 1;7:e40237. doi: 10.2196/40237 (PMC9932871; doi:10.2196/40237)

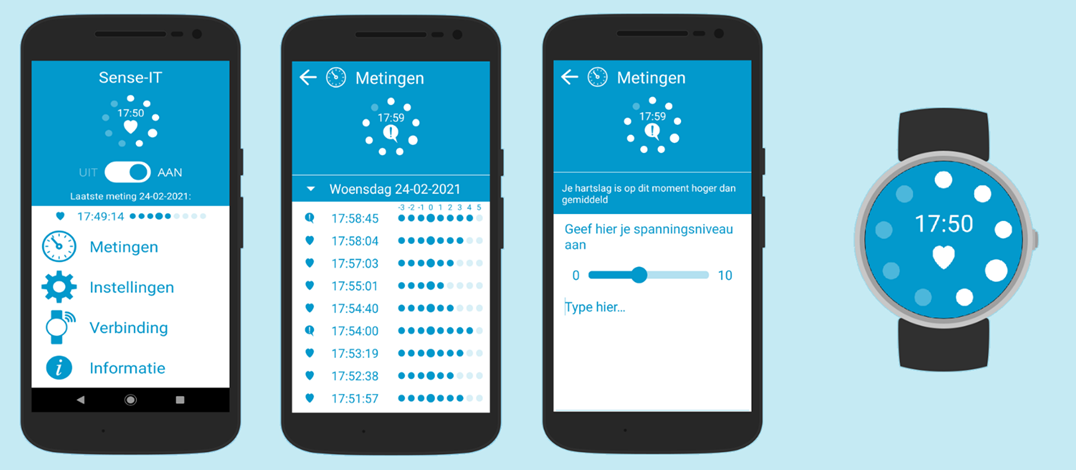

Supplement: Multimedia Appendix 1 [file formative_v7i1e40237_app1.png]
