# Supplementary material for: Forensic Psychiatric Outpatients’ and Therapists’ Perspectives on a Wearable Biocueing App (Sense-IT) as an Addition to Aggression Regulation Therapy: Qualitative Focus Group and Interview Study
Source: JMIR Form Res. 2023 Feb 1;7:e40237. doi: 10.2196/40237 (PMC9932871; doi:10.2196/40237)
Supplement: Multimedia Appendix 2 [file formative_v7i1e40237_app2.docx]

**Questioning route**

Key questions and topics to be addressed:

1. What could be the main added value of implementing the Sense-IT app in treatment, in your opinion?

*[Topics to be addressed if they do not emerge]*

- 1. *For patients*
  2. *For therapists*
  3. *For (the efficacy of) treatment*

*[Optional topic, if sufficient time: For what type of patient, or in which phase in therapy do you expect that implementing the Sense-IT app would be most valuable?]*

1. What do you see as the main barriers to implementing the Sense-IT app in treatment?

*[Topics to be addressed if they do not emerge]*

- 1. *Related to the Sense-IT app itself*
  2. *On the patient level*
  3. *On the therapist level*
  4. *On the organizational level*

*[Optionally, if they do not emerge: specifying subcategories of the organizational level]*

1. What do you need to get started with the Sense-IT app in treatment? Name one to three facilitators.

*[Optionally, if this topic not emerged: Do you think that, as a therapist, you should have tried the Sense-IT app yourself first?]*

1. Do you have any other recommendations that might promote the implementation of the Sense-IT app in treatment?
